# Omental evisceration in a gravid woman following second-trimester laparoscopy: A case report

**DOI:** 10.1016/j.crwh.2020.e00283

**Published:** 2020-12-26

**Authors:** Elizabeth A. Dilday, Regan L. Allen, Sarah A. Manning, Lisa Chao

**Affiliations:** a Department of Obstetrics and Gynecology, Parkland Health and Hospital System, United States of America; b University of Texas Southwestern Medical Center, Dallas, TX, United States of America

**Keywords:** Adnexal mass, Evisceration, Laparoscopy, Pregnancy

## Abstract

Surgeons performing laparoscopy in pregnancy are developing specific practices to improve care of gravid women. In this case, a pregnant 26-year-old patient underwent laparoscopic ovarian cystectomy in the second trimester, with cyst wall removal through an 8 mm umbilical port site incision. She returned four days later with pain and drainage from the umbilicus, and examination under anesthesia revealed omental evisceration through the umbilical incision. The patient healed well following excision of affected omentum, reapproximation of fascia in a simple, interrupted fashion, and closure of skin in a subcuticular fashion. Due to increased intra-abdominal pressure associated with a gravid uterus and other factors, port site closure for incisions smaller than 10 mm may improve healing and prevent complications of laparoscopic surgery in pregnancy.

## Highlights

• Laparoscopic surgery is the preferred modality for adnexal surgery in pregnancy
• Evisceration is an exceedingly rare complication following laparoscopy
• We report a case of omental evisceration through the umbilicus presenting days after second trimester laparoscopic cystectomy
• Fascial closure of incisions <10 mm may avoid laparoscopy complications in pregnancy

## 1 Introduction

While pregnancy was once considered to be a contraindication to laparoscopy, laparoscopic surgery is now a safe and acceptable alternative to open surgery in pregnancy [1]. Laparoscopy in pregnancy is associated with faster recovery times, shorter hospital stays and fewer wound infections compared to open surgery [1]. Laparoscopy is the preferred modality for adnexal surgery in pregnancy, with well-documented safety and a lower rate of preterm contractions compared to open surgery [2]. Symptomatic ovarian cysts have an incidence of 2.3–8.8% in pregnancy, and the incidence of adnexal masses requiring surgery in pregnancy is reportedly 1–2.3% of all gestations [3]. Progression to ovarian torsion can cause loss of ovarian function if not treated promptly [3].

While the safety of laparoscopy in pregnancy is well-documented, miscarriage, preterm labor and perinatal death are seen postoperatively in rare cases [2]. An uncommon complication of laparoscopy is port-site hernia, with reported incidence of 1.9% and 3.2% at two and five years after surgery, respectively [4]. Evisceration at the incision site after laparoscopy is extremely rare, especially during pregnancy.

As physicians gain experience with laparoscopy in pregnancy, modifications in techniques can improve care for gravid women. This report describes a woman in her second trimester of pregnancy with postoperative omental evisceration from the umbilical incision and makes recommendations for guidelines for fascial closure in pregnancy.

## 2 Case Presentation

**Fig. 1:**
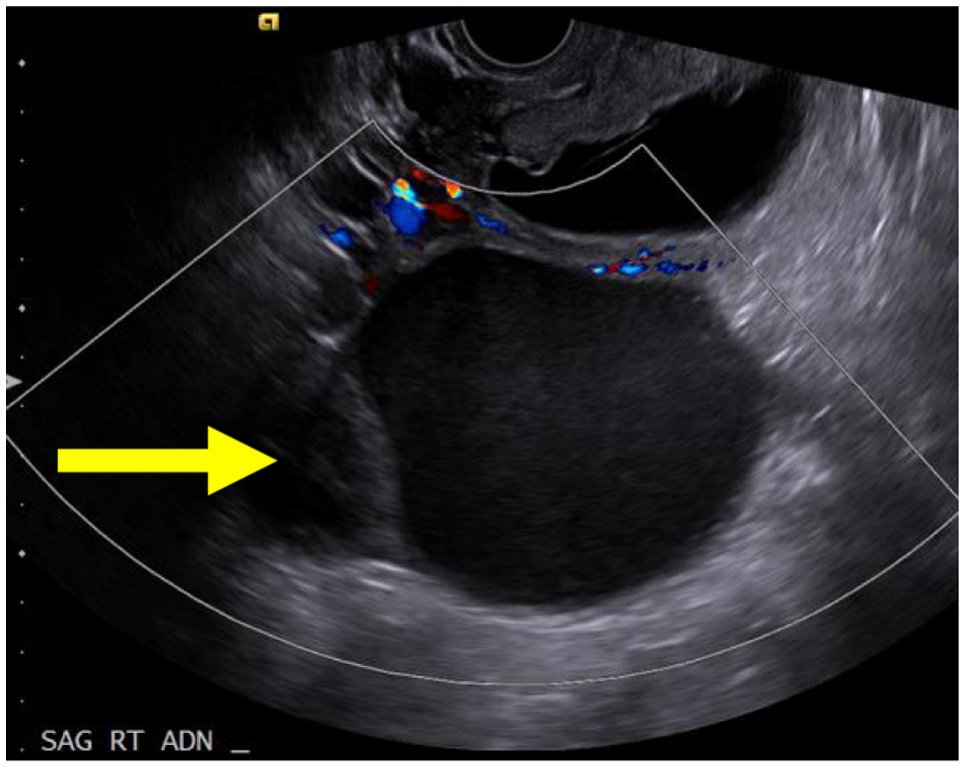
12.7 × 8.8 × 9.7 cm right adnexal cystic mass.

A 26-year-old woman (gravida 3 para 0) at 14 weeks 2 days of gestation presented with three weeks of intermittent right lower quadrant abdominal pain, nausea and emesis. She had no prior surgical history and no pertinent medical history other than overweight body habitus (body mass index 26.6 kg/m^2^). Pelvic ultrasound revealed a single live intrauterine pregnancy and a 12.7 × 8.8 × 9.7 cm right adnexal cystic mass (Fig. 1).

A preoperative diagnosis of right ovarian torsion was made and the decision was made to proceed with laparoscopy due to acute worsening of symptoms, desire for future childbearing and high index of suspicion for a surgical emergency. Three 5-mm incisions were initially used with intraperitoneal entry obtained at Palmer's point, a surgical landmark in the left upper quadrant of the abdomen, approximately 3 cm below the costal margin in the midclavicular line. Two additional incisions were placed in the right and left lower quadrants to accommodate laparoscopic ports. Intraoperative findings were notable for a 9 cm right ovarian cyst, and a right ovarian cystectomy was performed. An additional 8 mm incision was made at the base of the umbilicus for removal of the cyst wall from the abdominal cavity. The fascial layer was not closed at any of the port sites. All ports were subsequently removed and skin incisions were closed in a subcuticular fashion using 4-0 Monocryl (monofilament synthetic absorbable suture) and secured with Dermabond (cyanoacrylate tissue adhesive). She was discharged to home postoperatively. Surgical pathology was consistent with a benign corpus luteum cyst, which is the most common mass observed in cases of torsion in pregnancy [3]. Because the patient was well into the second trimester, she did not receive supplemental progesterone following removal of the corpus luteum.

**Fig. 2:**
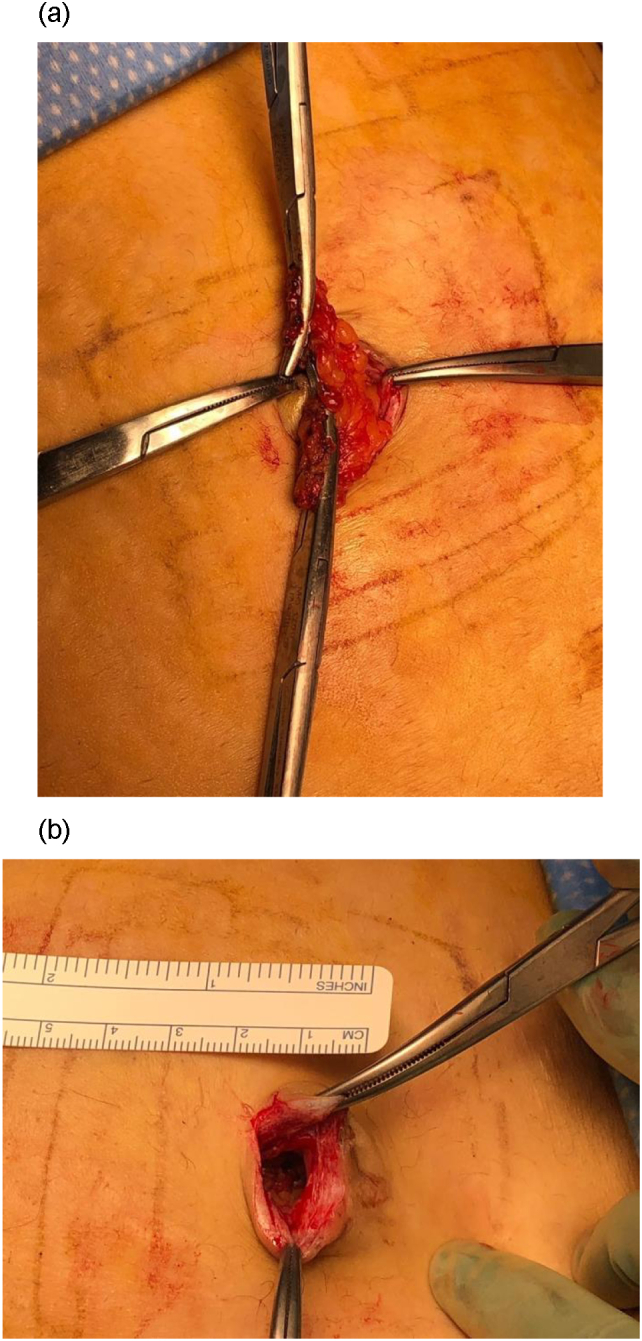
A. Omentum eviscerating at umbilical port site incision. B. Fascial defect measuring approximately 1 cm.

On postoperative day 4, the patient returned to the emergency room reporting a protruding mass and clear drainage from the umbilicus that began after a coughing episode. She was afebrile and noted return of normal bowel function. Examination revealed 1.5 cm of flesh-colored tissue protruding from the umbilicus. The patient was intolerant of bedside exploration and was consented for surgical management. Intraoperatively, there was a palpable 1 cm fascial defect, and the flesh-colored tissue was identified as evisceration of omentum (Fig. 2A,B).

**Fig. 3:**
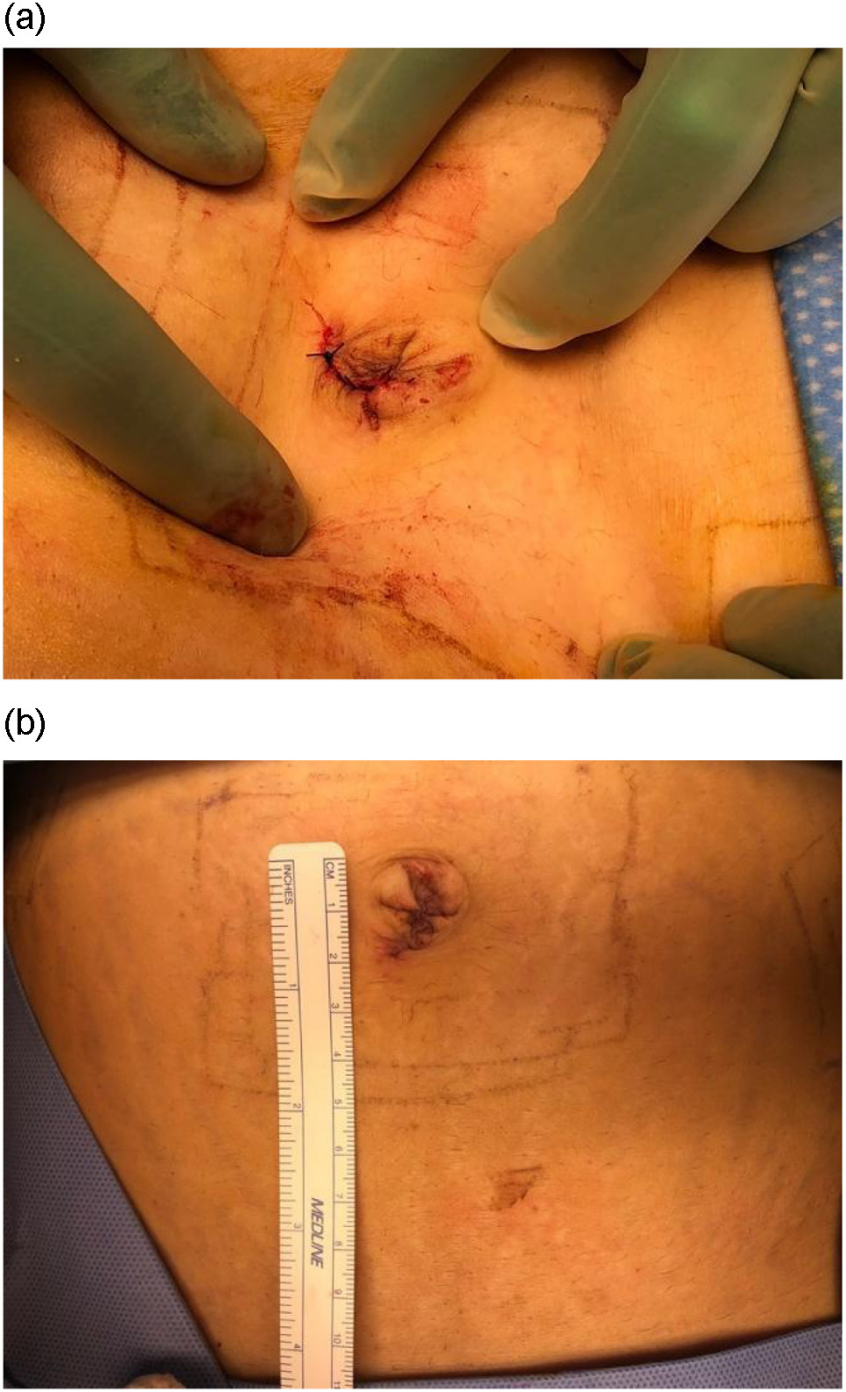
A. The fascial defect was reapproximated using 2–0 PDS suture in a simple, interrupted fashion. B. The skin was closed using 4-0 Monocryl.

Approximately 1 cm of strangulated omentum was excised and sent to pathology, and the remaining well-vascularized omentum was returned to the abdomen. The fascia at the umbilicus was reapproximated with three 2–0 PDS sutures (polydioxanone sterile synthetic absorbable monofilament suture) in a simple, interrupted fashion (Fig. 3A) and the skin incision was closed using 4-0 Monocryl (monofilament synthetic absorbable suture) in a subcuticular fashion (Fig. 3B). Final surgical pathology revealed omentum with acute inflammation. She recovered quickly and the umbilical incision healed well. The patient underwent an uncomplicated normal spontaneous vaginal delivery at 35 weeks of gestation after preterm prelabor rupture of membranes.

## 3 Discussion

This case highlights an unusual complication after laparoscopic surgery during pregnancy. Omental evisceration is exceedingly rare after laparoscopy, with an incidence rate of trocar site herniation of 1.5–1.8% [5]. Omental evisceration has been cited in the literature at incision sites as small as 5 mm in pediatric patients [6]. This patient's complication raises questions about best practices for laparoscopy when performed in pregnant patients, including alterations to guidelines for fascial closure for port site incisions smaller than 10 mm when performed in the setting of pregnancy.

The incidence of incisional hernia following laparoscopy, based on a survey by the American Association of Gynecologic Laparoscopists, is 21 per 100,000 in 4,385,000 procedures performed [7]. While uncommon, trocar site incisional hernias are a known complication of laparoscopic surgery and risk increases as a function of fascial incision size [7]. In addition, placement of trocars at the base of the umbilical ring may predispose to postoperative weakening due to the thin abdominal wall in the area [8]. If herniation occurs, it is likely to take place at least 36 h after surgery [8], as seen in this case.

Traditionally, larger port sizes, increased trocar movement, and longer surgeries were identified as indications for fascial closure [9]. There is no consensus whether or not fascia at trocar sites less than 10 mm should be closed, and there are no pregnancy-specific recommendations for fascial closure in laparoscopy [9]. According to a 2011 review by Yamamoto et al., fascial closure of 5 mm ports may be considered in cases where prolonged manipulation has occurred, as the initial incision may have extended with stretching [10]. The review concluded that choice should be left to the discretion of the surgeon [10]. For this patient, stretching of the fascial incision that occurred at the time of cyst wall removal from the abdomen likely contributed to hernia formation.

With the increased intra-abdominal pressure in pregnant patients compared to nonpregnant individuals, laparoscopic incisions are under greater tension in pregnancy and can be more likely to dehisce. Increased intra-abdominal pressure from a growing gravid uterus may predispose pregnant women to dehiscence and herniation from incisions smaller than 10 mm. It is possible that physiologic increases in estrogen and progesterone could contribute to tissue laxity. The patient described here also reported coughing prior to development of the hernia, which further increases intra-abdominal pressure.

Should we consider universal closure of laparoscopic port sites in pregnant women? The benefits of this strategy must be weighed against the drawbacks. Obstacles such as increased operating times (which can affect both the patient and her developing fetus), risks of suturing under closed technique (such as inadvertently suturing omentum and viscera to the abdominal wall), and challenges of using fascial closure devices near the gravid uterus (such as uterine perforation or injury to surrounding viscera and vessels), especially later in gestation, must be considered.

Other tactics to prevent herniation when operating on pregnant patients are more feasible and less risky. One strategy is placement of stay sutures in the fascia prior to trocar insertion; at the conclusion of the procedure, the sutures can be tied together after digitally probing the site to ensure that it is free of any contents from the peritoneal cavity [6]. A *Z*-shaped pathway for port insertion is another strategy to reduce herniation. With this “Z track technique,” the cannula is inserted into skin at a right angle, then reoriented horizontally after subcutaneous tissue is entered and angled toward pelvic brim; finally, the cannula is positioned perpendicularly for entering the peritoneal cavity [6,7]. Additional strategies to prevent herniation are use of the suction-irrigator to evacuate pneumoperitoneum to decrease the iatrogenic rise in intra-abdominal pressure, avoidance of significant coughing or Valsalva at time of extubation and application of an abdominal binder in the early postoperative period for pregnant patients to limit variation in intra-abdominal pressures due to mechanical compression [7].

## 4 Conclusion

It is unreasonable to draw universal conclusions about laparoscopy in pregnancy from one report that highlights a rare outcome. General strategies and considerations exist for performing laparoscopic surgery in pregnancy. Several techniques to prevent herniation and evisceration have been described in the literature, and attention to these techniques is especially warranted when operating during pregnancy. There are no widely-accepted guidelines for laparoscopic port site closure in pregnant patients based on size and location of the incision. However, this case makes one consider fascial closure in pregnant patients for incisions less than 10 mm due to increased intra-abdominal pressures and other factors. We recommend fascial closure if it is safe to carry out and if other factors predisposing to herniation such as pregnancy and obesity coexist. This is an area that warrants further study, but given the potential downsides of universal fascial closure, fascial closure in pregnancy should be carried out on a case-by-case basis.
